# Safety-Net FoodRx: Repeat Attendance and Produce Consumption Patterns in a Produce Distribution Program

**DOI:** 10.3390/nu18172884

**Published:** 2026-09-03

**Authors:** Frederick Ferguson, Michael Baham, William J. McCarthy, Arleen F. Brown, Alejandra Casillas, Stefanie Vassar, Gloria Moon, Julia I. Caldwell, Fatinah Darwish-Elsherbiny, Dipa Shah, Tony Kuo

**Affiliations:** 1 Department of Family Medicine, David Geffen School of Medicine at UCLA, 10833 Le Conte Avenue, 50-074 CHS, Los Angeles, CA 90095, USA; 2 Department of Health Policy and Management, UCLA Fielding School of Public Health, 650 Charles E. Young Dr. South, Suite 31-269 CHS, Los Angeles, CA 90095, USA; wmccarth@ucla.edu; 3 Division of General Internal Medicine and Health Services Research, Department of Medicine, David Geffen School of Medicine at UCLA, 200 Medical Plaza, Suite 420, Los Angeles, CA 90095, USA; 4 Nutrition and Physical Activity Program, Division of Chronic Disease and Injury Prevention, Los Angeles County Department of Public Health, 3530 Wilshire Blvd., Suite 800, Los Angeles, CA 90010, USA; 5 Department of Epidemiology, UCLA Fielding School of Public Health, Box 951772, Los Angeles, CA 90095, USA; 6 Population Health Program, UCLA Clinical and Translational Science Institute, 10833 Le Conte Ave., BE-144 CHS, Los Angeles, CA 90095, USA

**Keywords:** produce distribution, proportion of produce consumed, attendance frequency at produce events, intercept survey, safety-net healthcare systems

## Abstract

**Background**: Free produce distributions at safety-net health center sites represent a promising strategy for mitigating food insecurity. Understanding how these services connect to participant outcomes is essential for optimizing food delivery to the community. **Objectives**: In this secondary analysis of Los Angeles County FoodRx Clinic’s Produce Distribution Program (PDP), we evaluated the association between attendance frequency and the proportion of produce consumed (“all” versus “less than all”) among returning participants, adjusting for socio-demographic, household, and program characteristics. Additionally, we sought to identify factors contributing to incomplete produce consumption. **Methods**: Data were collected using intercept surveys at 16 healthcare sites hosting produce distributions from July to October 2024. Among 451 surveyed participants, 368 were repeat attendees who had visited the distribution events more than once in the last 12 months. **Results**: Among repeat attendees, 64% reported their household consumed all produce received, while 36% consumed only a portion. Higher attendance frequency (4+ times vs. 2–3 times) was associated with significantly higher odds of all produce consumed (adjusted odds ratio: 2.38, confidence interval: 1.43–3.95). **Conclusions**: These findings provide critical insights for refining PDP operations and minimizing incomplete produce consumption among repeat attendees of this program.

## 1. Introduction

Food insecurity and lack of access to fresh produce contribute to health disparities among low-income and ethnically diverse populations across Los Angeles County (LAC) [1,2,3]. Lack of access to fresh produce can lead to poor diet quality, which is an established risk factor for major chronic health conditions such as diabetes, cardiovascular disease, and certain cancers [1,3,4,5,6,7,8,9,10,11]. Programs that address food insecurity and increase access to healthy food options represent critical public health practices, as they provide essential services that help mitigate diet-related chronic diseases, particularly among populations served by safety-net healthcare systems. In the Los Angeles area context, the construct “safety-net” refers to a system or systems of health and human services that provide significant uncompensated care to mostly low-income, historically minoritized populations with limited English proficiency.

Safety-net clinics and community-based food distribution programs are frontline entities that screen for food insecurity and deliver coordinated care to meet patients’ nutritional and medical needs [7]. Previous research has shown that produce distribution programs (PDPs) can increase fruit and vegetable consumption, improve cardiometabolic health, and reduce overall medical spending [3,5,6,12,13,14]. How patients can effectively consume the free distributed produce to its fullest (versus [vs.] partially consuming it) is not well understood in the existing scientific literature. Nor is there consensus about which patients could benefit the most from participating in PDPs. A similar gap in knowledge exists regarding active participation in public assistance programs like the Supplemental Nutrition Assistance Program (SNAP), as well as the use of medically tailored diets and food-as-medicine interventions [11,13,15,16].

Contextual factors may influence whether individuals consume all or only part of the produce they receive through PDPs. Structural barriers, such as limited time for food preparation, inadequate cooking equipment or food storage, and receipt of quantities of produce that exceed household capacity, may all contribute to incomplete consumption [17,18,19]. Additionally, food preferences and familiarity with certain fruits and vegetables may influence produce consumption [20,21].

A related concept is food neophobia, defined as the fear or reluctance to try unfamiliar foods, which can shape individuals’ willingness to experiment with new produce items [22,23]. Food neophobia has been associated with reduced dietary diversity, poorer diet quality, and increased risk of adverse health outcomes, including obesity, metabolic disorders, and eating disorders such as avoidant/restrictive food intake disorder [22,23,24,25]. While food neophobia is often more pronounced in early childhood, evidence suggests it can persist into adulthood, especially in individuals with low educational attainment [26]. Prior studies indicate that the average age of participants in FoodRx PDPs is approximately 57 years, highlighting the need to understand how this demographic engages with and consumes distributed produce [3]. While these program interventions have been shown to increase access to healthy food options, participant consumption of the distributed produce to achieve targeted nutritional benefits remains under-characterized.

Despite potential barriers to full implementation, the LAC FoodRx Clinic’s PDP (“FoodRx Clinic”) represents an efficient model for addressing food insecurity in the region. Designed as a public–private partnership (learning network) among the Los Angeles County Department of Public Health (DPH), the Los Angeles County Department of Health Services (DHS), local safety-net clinics, community-based organizations, and regional food bank partners, the FoodRx Clinic aims to expand access to healthy food by regularly distributing free, culturally relevant fruits and vegetables to patients at multiple free clinic sites across the county. Prior research has shown that a majority of FoodRx Clinic attendees lived in food-insecure homes with a personal or family history of chronic disease [3,7]. Attendance at FoodRx Clinic distribution events has been found to be associated with higher consumption of fruits and vegetables, suggesting that repeat attendance and exposure may encourage healthier eating habits over time [3]. However, despite promising results, this seminal work did not investigate whether participating families consumed all the distributed produce or if a significant portion reported incomplete produce consumption for their household.

In this study, we address these gaps about the PDP by evaluating how the frequency of attendance at the FoodRx Clinic is related to the completeness of produce consumption among participants. Our primary objective was to describe the association between attendance frequency and the proportion of produce consumed (all vs. less than all), adjusting for socio-demographic, household, and program characteristics. We hypothesize that frequent attendance is positively associated with a higher proportion of produce consumed. Our secondary objective was to identify factors that may have contributed to the reported incomplete produce consumption. We hypothesize that structural barriers—such as high household demands, storage limitations, or competing resource constraints—are associated with incomplete produce consumption, resulting in a lack of perceived benefit from the PDP. Data on these consumption patterns are intended to inform program refinements and efforts to increase complete produce consumption of produce received from the FoodRx Clinic.

## 2. Materials and Methods

### 2.1. Setting and Context

The Los Angeles County FoodRx Collaborative, formed in March 2021 by the Departments of Public Health and Health Services, works to reduce diet-related chronic diseases by increasing access to healthier food within safety-net healthcare sites. This Collaborative seeks to reduce food insecurity among patients and community members and improve clinical outcomes by coordinating PDP distributions, alongside nutrition education and referrals to SNAP (known as CalFresh in California). Between October 2020 and March 2025, 16 DHS and DPH health center sites facilitated a total of 944 onsite produce events, distributing more than 4 million pounds of free produce to 831,903 people (217,791 households). At the time of data collection in 2024, FoodRx PDP distributions were regularly occurring at each of the 16 safety-net health center sites, with 1 to 2 PDP distributions being hosted each month.

### 2.2. Data Collection

This secondary analysis of survey responses utilized data from a cross-sectional study jointly conducted by the LAC FoodRx Collaborative and the University of California, Los Angeles (UCLA), National Institutes of Health/National Heart, Lung, and Blood Institute’s Disparities Elimination through Coordinated Interventions to Prevent and Control Heart and Lung Disease Risk (DECIPHeR) Alliance; the dataset is described elsewhere [3]. Data collection took place between July and October 2024; intercept surveys were administered to adults who attended free produce distribution events hosted at 16 DHS and DPH safety-net health center sites. PDP event attendees were approached by evaluators who were onsite for each particular day; most prospective participants were waiting in line for the produce distribution. Due to the scale of these events, evaluators simultaneously approached attendees throughout the distribution line to capture a diverse and comprehensive sample. As the events were open to the public, attendees included both community members and patients referred to these events by their healthcare providers. To satisfy study eligibility, staff confirmed (on paper) that selected participants were over the age of 18, resided in Los Angeles County, and had not completed the survey previously. Prior to starting the questionnaire, they were informed individually that the survey was voluntary and participation would not affect their ability to receive free produce or other services at their usual care site. The questionnaire was developed by DPH-DECIPHeR evaluators using adapted questions from prior surveys, supplemented by internally developed items focused on FoodRx Clinic’s specific operational attributes. The questionnaire did not undergo formal pilot testing or content validation because of the project timeline and available staff capacity. Several items, including prior PDP attendance frequency and consumption of distributed produce, were adapted from surveys previously fielded at other free produce distribution sites in Los Angeles County [27,28]. Program-specific items were developed internally for the present evaluation. Food insecurity was assessed using the validated U.S. Department of Agriculture six-item Food Security Module. The survey was available in English and Spanish and self-administered using pen and paper. All participants gave informed consent prior to starting the survey. Those who completed it were compensated for their time with a $15 grocery gift card.

To provide operational perspectives about the distributions, event-level data were also collected from clinic staff who ran the events. Across the 16 sites, from July to October 2024, an estimated 4032 households attended the PDP distributions, representing approximately 14,770 individuals who consumed the distributed food within those households. During this period, clinics reported receiving approximately 71,335 pounds of produce from food distribution partners, of which about 68,558 pounds were distributed to event attendees. The surveys were administered during the summer and early fall of 2024—a seasonal timeframe when produce availability and variety are typically higher. As such, this may have influenced the range of fruits and vegetables distributed at these events. Reported fruit categories included stone fruits (e.g., peaches and nectarines), berries (e.g., grapes, strawberries, and blackberries), citrus fruits, melons, pome fruits such as apples, and tropical fruits including mango and papaya. Vegetable distributions included fruiting vegetables (e.g., tomatoes, squash, and cucumbers), root and tuber vegetables (e.g., potatoes and carrots), cruciferous vegetables (e.g., broccoli and cauliflower), leafy greens (e.g., lettuce and bok choy), alliums (e.g., onions), legumes (e.g., green beans), and other seasonal items such as corn and mushrooms. Clinic staff generally reported high produce quality; 87.5% rated the produce as either “excellent” or “good” at these events. These reports by staff provided rich context regarding the quantity, quality, and diversity of the distributed produce, and offered critical insights into consumption barriers and the primary reasons households were unable to consume all the items received.

### 2.3. Outcomes

Our primary outcome was the proportion of distributed produce consumed—i.e., fruits and vegetables received from one or more of the events hosted by the FoodRx Clinic. This outcome was assessed using the survey question, “How many of the fruits and vegetables do you typically eat from the produce you receive from these events?”, with responses “All”, “Most”, “Half”, “A few”, “None”, and “Participant did not answer.” For modeling purposes, the outcome was further dichotomized into “All Produce Consumed” and “Less than All Produce Consumed,” in alignment with our primary objective of understanding the different associations between these two groups. Complete consumption was selected as the primary outcome because it represents the most optimal impact of the PDP and was, therefore, considered the most programmatically meaningful endpoint.

Our secondary outcome was a question about reasons for incomplete produce consumption, as assessed by the survey question, “Why did your household not eat all of the produce from these events? (Check all that apply).” Response options included predefined categories—“All of the produce was eaten,” “I received too much produce,” “I did not like the produce that was given out,” “The produce was not fresh,” “The produce went bad before I could eat it,” “I didn’t have adequate cooking equipment or ways to store food,” “I didn’t know how to cook and/or include the produce in my meals,” and “I didn’t have time to cook”—as well as an open-ended “Other” option. Because this was a check-all-that-apply item stored as a string variable, responses to the “Other” option were operationalized into dummy variables (0 = No, 1 = Yes) using keyword string matching in Stata. Conceptually related responses were grouped into analytic categories: food quality issues (did not like produce, not fresh, or went bad), knowledge or equipment barriers (lack of cooking/storage capacity or knowledge), too much produce, and not enough time. Since participants could select multiple responses, categories (other than “All produce eaten”) were not mutually exclusive.

### 2.4. Independent Variable

Our primary predictor or independent variable was the attendance frequency at PDP events in the last 12 months. This was assessed using the survey question, “In the last 12 months, how many times have you (or anyone in your household) ever come to Produce Distributions at this location?,” with the responses, “First time”, “2–3 times,” “4–5 times,” “6–7 times,” “8–9 times,” “10–11 times,” “12 times or more,” and “Participant did not answer”. For our analysis, prior attendance was categorized as 2–3 versus ≥4 visits to distinguish participants with limited prior exposure from those with more sustained engagement at the site (≥4 visits represent quarterly or more frequent attendance). First-time attendees were excluded from the primary analyses examining prior attendance because they had no previous produce distribution experience and, therefore, had little basis for reporting their “typical” experience.

### 2.5. Covariates

Study covariates comprised both individual- and household-level characteristics. Those at the individual level included: gender (female or male); age (18–44, 45–64, or ≥65); and Hispanic origin (yes or no). Those at the household level included: household number (picking up produce for one household or more than one household); persons in the household (1–2 people or more than two people in the household); SNAP participation—enrolled and participating (yes or no); and overall number of assistance programs in which the individual currently participates (none, one, or two or more). Response options for assistance programs included: SNAP/CalFresh/Food Stamps; Medi-Cal; Special Supplemental Nutrition Program for Women, Infants, and Children (WIC); CalWorks (Temporary Assistance for Needy Families); Housing Assistance Programs (Section 8); Social Security Disability Insurance (SSDI); and Supplemental Security Income (SSI). Food insecurity was assessed using the United States Department of Agriculture (USDA) six-item Food Security Module. Based on the scores assigned to these module responses, a raw score of 0–1 was coded as high or marginal food security, a raw score of 2–4 was coded as low food security, and a raw score of 5–6 was coded as very low food security. Attendees who participated in the survey were asked if they or anyone in their household were diagnosed with the following health conditions: heart disease, prediabetes, diabetes, hypertension (high blood pressure), or overweight/obesity. Chronic condition burden was categorized into the following groups: those reporting none, one, or two or more chronic conditions.

### 2.6. Analyses

All analyses were conducted using Stata Version 19 (StataCorp LLC, College Station, TX, USA). Study protocols, including the survey questionnaire, were reviewed and approved by the Institutional Review Board at the University of California, Los Angelesprior to field administration.

Frequencies and percentages were generated to display descriptive statistics. Bivariate analyses with chi-square testing were performed to test distributional differences between participants reporting all produce consumed and those reporting less than all produce consumed, adjusting for attendance frequency and various covariates (Appendix A: Table A1 and Table A2). Multivariable regression analyses examined associations between attendance frequency and the primary outcome of interest: proportion of produce consumed. These multivariable logistic regressions adjusted for the independent variable, several socio-demographic characteristics, and other covariates. Model results are presented as odds ratios (ORs) or adjusted odds ratios (AORs), with corresponding 95% confidence intervals (CIs).

To assess the models’ robustness, we conducted sensitivity analyses that evaluated alternative model specifications, such as removing overlapping measures of public assistance, redefining chronic conditions to exclude overweight/obesity, restricting analyses to participants with multiple event attendances, and testing interaction terms between attendance frequency and SNAP participation. Missingness was integrated into the models as a separate covariate category; results remained unchanged after accounting for this variable. Model fit was assessed using likelihood ratio tests and pseudo R^2^ statistics.

## 3. Results

### 3.1. Participant Characteristics and Produce Consumption

Of the 497 participants in the original survey sample, 45 had incomplete surveys and one was under the age of 18, leaving the final sample size at *n* = 451. For our independent variable (attendance frequency), after excluding first-time attendees, the final sample size was *n* = 368.

A total of 451 participants reported household produce consumption, with 368 indicating repeat attendance (i.e., attending the events more than once) in the last 12 months. Most survey participants were female (76%), with an average age of 57 years; age range: 19–89. A majority identified as Hispanic/Latino (83%). Individual-level demographic characteristics, including gender, age group, and Hispanic/Latino origin, were not significantly associated with whether households consumed all produce received from the FoodRx Clinic (all *p* > 0.05).

**Figure 1:**
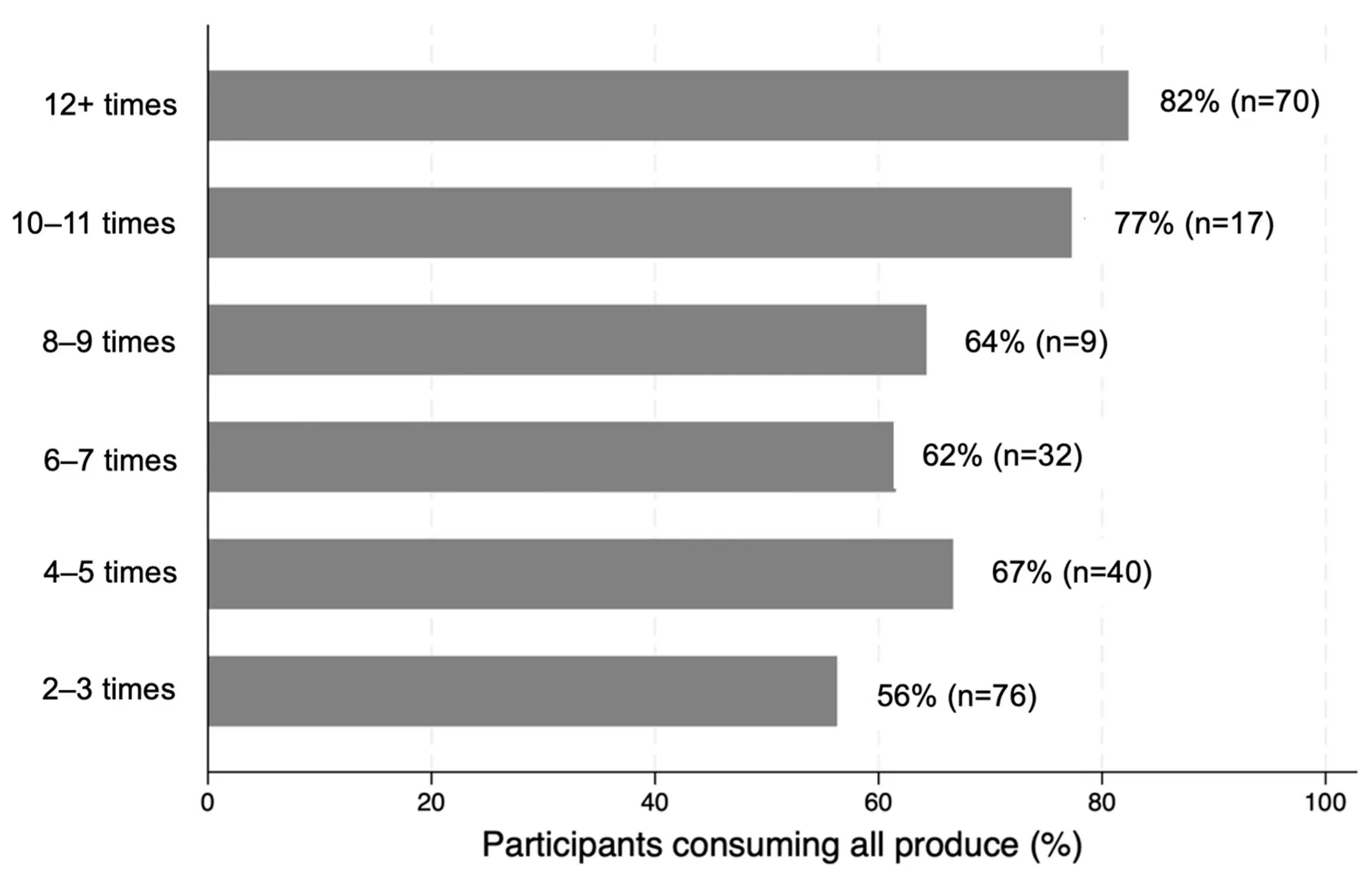
Percentage of participants consuming all distributed produce by attendance frequency.

Figure 1 shows the proportion of distributed produce consumed by attendance frequency. Bivariate analyses across all attendance frequency categories are presented in Table 1 and Appendix A; these analyses included individual-level demographic characteristics, household-level characteristics, and the outcome variable. As attendance frequency increased, a higher proportion of participants reported that their households consumed all the produce received.

Overall, of those with repeat attendance, 64% reported their household consumed all the produce received from the PDP events, while 36% reported their household consumed less than all distributed produce (Table 1). Increased attendance frequency (4+ times vs. 2–3 times) was significantly associated with consuming all produce. Among those who consumed all the produce, 69% had attended 4 or more times; among those who consumed less than all the produce, only 52% had attended 4 or more times (chi-square (1) = 9.5589; *p* = 0.002).

Household-level characteristics showed several notable patterns. A higher proportion of households with more than two individuals reported that their household consumed all distributed produce compared with smaller households (one or two people) (chi-square (1) = 9.2734; 82% vs. 69%, *p* = 0.002). Food insecurity was common in the sample, with approximately half reporting low or very low food security; however, food security status was not significantly associated with produce consumed (chi-square (1) = 1.4685; *p* = 0.226). Similarly, SNAP participation and the number of assistance programs used were not significantly associated with produce consumed. Participants reported a high burden of chronic health conditions overall. Nearly half reported hypertension (49%); 47% reported diabetes, borderline diabetes, or prediabetes; and 23% reported overweight or obesity. Overall chronic condition burden or presence of chronic conditions was not significantly associated with produce consumed.

### 3.2. Attendance Frequency and Produce Consumption

Table 2 shows the multivariable logistic regression model analyses. In the unadjusted model, participants who attended produce distribution events four or more times in the previous 12 months had significantly higher odds of reporting all produce consumed compared with those who attended 2–3 times (aOR = 2.01, 95% CI: 1.29–3.13). This association remained significant after adjusting for individual-level characteristics, including gender, age, and race/ethnicity (Model 2: aOR = 2.00, 95% CI: 1.28–3.13). When household-level characteristics were introduced (Model 3), the association strengthened slightly (aOR = 2.34, 95% CI: 1.41–3.87). In the fully adjusted model, accounting for both individual- and household-level characteristics, frequent attendance was significantly associated with increased odds of reporting all distributed produce consumed (aOR = 2.38, 95% CI: 1.43–3.95). Among household-level covariates, SNAP participation was associated with lower odds of reporting all distributed produce consumed (aOR = 0.46, 95% CI: 0.26–0.83). Other variables—including gender, age, race/ethnicity, household size, food insecurity, and chronic disease burden—were not significantly associated with all produce consumed in the fully adjusted model. 

### 3.3. Reasons for Not Consuming All Distributed Produce

Among survey participants who reported that their household did not consume all produce received from the PDP (*n* = 180), several reasons were identified for the incomplete consumption (Table 3). The most stated reason was food quality concerns, including that the produce was not fresh, spoiled before consumption, or that they did not like the items (21.7%). Knowledge and equipment barriers—such as not knowing how to prepare certain produce offerings or lacking adequate cooking equipment or food storage—were reported by 13.9% of the participants. Receiving too much produce relative to household capacity to consume it was reported by 11.1%, while a lack of time to cook or prepare food was reported less frequently (3.9%).

Because survey participants were able to select multiple responses in the survey, the reasons for not consuming all the produce received were not mutually exclusive. Our findings suggest that both structural barriers (e.g., preparation time, equipment) and misperceptions of produce quality or familiarity may have influenced how attendees consumed foods received from the FoodRx Clinic events.

## 4. Discussion

These findings provide insights into how produce distributed by the FoodRx Clinic are consumed by attendees. Survey participants who attended events four or more times in the last 12 months had higher odds of consuming all produce received when compared to those who attended only 2–3 times, even after adjusting for individual- and household-level characteristics. More sustained engagement was associated with increased odds of complete produce consumption, suggesting that the potential role of continued participation in supporting produce consumption and longer-term dietary behavior change warrants prospective investigation. One explanation is that frequent attendance reinforces the routine incorporation of fruits and vegetables into household meals and improves attendees’ capacity to plan and prepare distributed foods. Additionally, repeated exposure to distributed produce may increase familiarity with available items and mitigate food neophobia—a frequently underappreciated psychological deterrent to consumption, particularly for minimally processed fruits and vegetables [29,30]; however, these putative mechanisms were not assessed in the present study.

The results contribute to a growing body of literature that supports food-as-medicine interventions as a standard component of medical treatment. Understanding how participants utilize these resources informs and guides innovative programming designed to prescribe produce as nutritional therapies [31,32]. This relationship is essential for ensuring that onsite food interventions are effectively translated into meaningful practice and supports that improve patient health outcomes.

Notably, attendees participating in SNAP had significantly lower odds of consuming all distributed produce. It is possible that SNAP recipients already purchase staple groceries through authorized retailers; thus, PDP produce acts as a supplemental rather than a primary food source. Within this context, distributed items complement existing household supplies without fully replacing purchased foods.

Alternatively, the findings may reflect structural and behavioral constraints disproportionately affecting lower-income households, including limited financial flexibility to experiment with new foods and heightened risk aversion to incomplete produce consumption [21]. In this setting, food neophobia is a hypothesized mechanism that could be particularly pronounced, as trying unfamiliar, minimally processed foods carries a perceived economic risk if the items are not accepted or consumed by household members [21,30,33]. Selection bias may contribute to this observation, as the analysis only included SNAP participants who chose to attend the FoodRx Clinic events—their socio-demographic profiles may differ from those who did not attend. We speculate that SNAP participants attending these events may have lower incomes and higher body mass indexes than non-attendees. In the existing literature, lower income is correlated with eating lower-quality, more affordable calorie sources—typically foods that are highly processed, fiber-poor, and calorie-dense [34,35].

### 4.1. Limitations

Several limitations should be considered when interpreting these findings. First, the study population was recruited from produce distributions serving safety-net healthcare clinic populations in Los Angeles County, and most participants were from Hispanic/Latino households. Therefore, the findings may not generalize to populations with different demographic or socioeconomic characteristics, from other geographic regions, or involving produce distribution programs implemented outside safety-net healthcare settings. Second, because this was a secondary analysis of data originally collected for program evaluation, our ability to assess additional contextual factors influencing consumption was constrained. Additionally, the program evaluation questionnaire did not undergo formal pilot testing or content validation, although several items were adapted from previously fielded surveys and food insecurity was assessed using the validated USDA six-item Food Security Module. Third, the cross-sectional design precludes establishing temporality or causality between produce distribution attendance and the totality of produce consumption. Participants who are already more inclined to consume fruits and vegetables may also be more likely to return to produce distributions; thus, reverse causation or self-selection is a legitimate alternative explanation for the observed association. Finally, while the analysis adjusted for available demographic and household characteristics, residual confounding could also explain the observed association. Factors such as cooking skills, nutrition knowledge, baseline fruit and vegetable intake, household income, transportation access, and food preparation practices may be associated with both program attendance and produce consumption, but they were not available in this secondary dataset.

The analytic sample was limited to returning attendees who voluntarily participated in the survey, introducing a risk of selection bias. Returning attendees may be more motivated, engaged with healthy eating, or satisfied with the PDP than non-returning participants, whom we were unable to survey. Consequently, findings may not be generalizable to all individuals who have ever participated in the PDP—particularly those who did not return to subsequent distributions. Prior produce distribution attendance and produce consumption were dichotomized to represent conceptually meaningful distinctions between limited and more sustained engagement (2–3 vs. ≥4 prior visits) and between incomplete and complete consumption of distributed produce, respectively. While these categorizations facilitated interpretation, they resulted in a potential loss of information across the original response categories, and may have reduced statistical power or obscured more nuanced associations. On the other hand, the dichotomized outcome was fully consistent with the distributional assumption of logistic regression, whereas the non-normal distribution of the frequency of PDP attendance was not consistent with the normal distribution assumption of ordinary least-squares regression. In any case, findings should be interpreted as associations between these defined groups rather than as evidence of dose–response relationships across the full range of attendance frequency or produce consumption. Additionally, produce consumption and related behaviors were self-reported, which introduces potential social desirability bias or recall bias—particularly as survey participants received a $15 produce gift card incentive for their participation in the survey study. Lastly, data collection occurred during the summer and early fall of 2024, a period of peak seasonal availability; consequently, the quality and variety of produce during the study may not reflect what is available in other periods.

Despite these limitations, our study provides valuable real-world insights into the operations of the FoodRx Clinic and other similar PDPs. These findings highlight opportunities for researchers and program implementers to learn and refine produce distributions as an efficient, scalable strategy for improving the diets of participating households.

### 4.2. Future Directions

Future research should build upon our findings by examining the specific barriers identified by survey participants who did not consume all the produce they received—namely, food quality concerns, limited preparation knowledge or equipment, storage challenges, cooking time constraints, food familiarity, and food neophobia. Longitudinal studies investigating these barriers are needed to determine the temporal and potentially causal relationships between repeated program attendance and the consumption of distributed produce. Additionally, exploring how participation in SNAP and other public food assistance programs influences consumption can inform the design of interventions that integrate more seamlessly within the broader U.S. food assistance landscape. Interventions pairing distributions with nutrition education and hands-on cooking demonstrations could be particularly effective in increasing the familiarity, confidence, and acceptability of minimally processed foods. Similarly, qualitative research capturing attendees’ perspectives on produce preferences and household decision-making can also offer deeper insights into how distributed foods are incorporated into daily meals.

At the systems level, analyzing the flow of produce—from food bank and community-based organization procurement to surplus diversion and onsite distribution—can identify leverage points to maximize food quality, variety, and quantity for low-income households in need. Addressing these challenges through client-centered design, culturally relevant offerings, and targeted education can enhance the efficiency of PDPs nationwide. Over time, reinforcing both programmatic and system-level approaches will amplify the impact of PDPs.

## 5. Conclusions

The FoodRx Clinic represents a promising strategy for improving healthy food access among populations at high risk of diet-related chronic diseases. Maximizing program impact requires more than just physical access; it necessitates ensuring that households can effectively plan, store, and prepare distributed foods. In this analysis, repeat attendance was significantly associated with greater produce consumption. Addressing the consumption challenges that may come with food and produce knowledge/familiarity will require refined programming, enhanced attendee education, and stronger coordination of the food procurement and distribution systems supporting these food-as-medicine initiatives. Integrating participant-informed operations, culturally relevant produce selection, and practical cooking and storage supports could be evaluated as strategies that help ensure distributed foods are effectively incorporated into household diets. Furthermore, strengthening PDP design and implementation fidelity may help increase the consumption of minimally processed, fiber-dense, and polyphenol-rich foods, ultimately improving diet quality and enhancing the management and prevention of diet-related chronic diseases.

## Figures and Tables

**Table 1:** Amount of produce consumed by participant characteristic.

Characteristic (Covariate)	Description	All Produce Consumed	Less Than All Produce Consumed	*p*-Value (Chi-Square) *
^a^ Total *n* = 451		*n* = 287 (64%)	*n* = 164 (36%)	
^b^ Attendance Frequency, *n* = 368	2–3 times	76 (31%)	59 (48%)	0.002
	4+ times	168 (69%)	65 (52%)	
Individual Characteristics				
Female		218 (76%)	123 (75%)	0.820
Age (mean: 57 years; range: 19–89 years)	19–44	47 (16%)	28 (17%)	0.449
	45–64	122 (43%)	60 (37%)	
	65+	118 (41%)	76 (46%)	
Hispanic Origin	Yes	237 (83%)	128 (78%)	0.239
^c^ Household Characteristics				
Food Security	High/Marginal/No Food Insecurity Reported	130	84	0.226
	Low/Very Low	157	80	
SNAP/CalFresh/Food Stamps	Yes	62 (22%)	41 (25%)	0.408
^d^ Number of Assistance Programs	0	69 (24%)	36 (22%)	0.852
	1	147 (51%)	88 (54%)	
	2 or more	71 (25%)	40 (24%)	
Household Number	1 Household	186 (65%)	101 (62%)	0.494
	>1 Household	49 (17.1%)	39 (23.8%)	
Persons in Household, *n* = 424	1 or 2 People	49 (18%)	47 (31%)	0.002
	>2 People	223 (82%)	105 (69%)	
High Blood Pressure	Yes	140 (49%)	79 (48%)	0.901
Heart Disease	Yes	29 (10%)	17 (10%)	0.930
Diabetes/Borderline Diabetes/Prediabetes	Yes	136 (47%)	68 (41%)	0.224
Overweight/Obesity	Yes	66 (23%)	27 (16%)	0.099
Chronic Condition Burden, *n* = 398	None	45 (18%)	32 (22%)	0.391
	1	92 (36%)	57 (39%)	
	2+	115 (46%)	57 (39%)	

^a^ Total *n* of households reporting produce consumption. Unless otherwise specified, the sample sizes for these descriptive characteristics or covariates are equal to the total sample size (*n*) of the study. ^b^ Attendance frequency at FoodRx Clinic produce distribution events in the last 12 months. ^c^ Survey questions asked if participants or anyone in their household had the characteristic(s). ^d^ Response options for assistance programs included: Supplemental Nutrition Assistance Program (SNAP)/CalFresh/Food Stamps; Medi-Cal; Special Supplemental Nutrition Program for Women, Infants, and Children (WIC); CalWorks (Temporary Assistance for Needy Families); Housing Assistance Programs (Section 8); Social Security Disability Insurance (SSDI); and Supplemental Security Income (SSI). * Chi-square test; statistical significance threshold: *p* < 0.05.

**Table 2:** Multivariable logistic regression models evaluating the association between produce distribution program attendance frequency and produce consumption. Outcome: produce consumed (all versus less than all). Primary Predictor: attendance frequency.

Variable	Model 1	Model 2	Model 3	Model 4
	OR (95% CI)	aOR (95% CI)	aOR (95% CI)	aOR (95% CI)
Attendance Frequency 4+ (ref: 2–3 times)	2.01(1.29–3.13)	2.00(1.28–3.13)	2.34(1.41–3.87)	2.38(1.43–3.95)
Female (ref: male)	—	0.92(0.55–1.53)	—	0.87(0.49–1.55)
Age 45–64 (ref: <45)	—	1.01(0.51–2.00)	—	1.00(0.47–2.12)
Age ≥ 65 (ref: <45)	—	0.84(0.43–1.64)	—	0.79(0.37–1.70)
Hispanic/Latino Origin (ref: non-Hispanic/Latino origin)	—	1.30(0.74–2.27)	—	0.98(0.51–1.88)
Household Size > 2 (ref: 1–2-person household size)	—	—	1.51(0.86–2.67)	1.48(0.82–2.68)
Household Number > 1 (ref: 1 household)	—	—	0.84(0.50–1.40)	0.98(0.51–1.42)
SNAP Participation (ref: no SNAP participation)	—	—	0.49(0.28–0.87)	0.46(0.26–0.83)
Food Insecure (ref: food secure)	—	—	1.45(0.88–2.40)	1.47(0.89–2.44)
1+ Chronic Condition Category (ref: none)	—	—	1.18(0.59–2.37)	1.20(0.59–2.44)
2 or more (ref: none)			1.17 (0.60–2.31)	1.21 (0.60–2.42)
*n*	368	368	307	307
Pseudo R^2^	0.020	0.023	0.058	0.060

Note: OR = odds ratio; aOR = adjusted odds ratio; CI = confidence interval. Model 1: Unadjusted association between attendance frequency and proportion of produce consumed. Model 2: Adjusted for individual characteristics (gender, age group, and race/ethnicity). Model 3: Adjusted for household characteristics (household size, household number, SNAP participation, food security status, and chronic conditions). Model 4: Fully adjusted model including individual- and household-level characteristics.

**Table 3:** Reported reasons for not consuming all the distributed produce among participants who did not consume all produce (*n* = 180).

Reason for Not Eating All Produce	*n*	% of Those Who Did Not Consume All Produce (*n* = 180)
Food quality issues (not fresh, spoiled, did not like produce)	39	21.67%
Knowledge or equipment barrier (lack of cooking/storage capacity or knowledge)	25	13.89%
Too much produce received	20	11.11%
Not enough time to cook	7	3.89%

Note: Percentages were calculated for participants who did not report eating all produce (*n* = 180). These categories are not mutually exclusive; survey participants may select more than one reason.

## Data Availability

Raw data from this project can be made available upon request for educational and other reasonable purposes. Please forward any request to the corresponding author.

## Abbreviations

The following abbreviations are used in this manuscript:

PDP	Produce distribution program
LAC	Los Angeles County
SNAP	Supplemental Nutrition Assistance Program
FoodRx Clinic	Los Angeles County FoodRx Clinic’s Produce Distribution Program

## Appendix A

### Appendix A.1

**Table A1:** Bivariate associations between attendance frequency and household demographic characteristics among returning users.

**Attendance Frequency**	**Sex**			
	**Female**		**Male**	***p*-Value**
2–3 times	98 (27%)		38 (30%)	0.608
4–5 times	47 (13%)		13 (10%)	
6–7 times	41 (11%)		11 (9%)	
8–9 times	11 (3%)		3 (2%)	
10–11 times	20 (6%)		3 (2%)	
12+ times	60 (17%)		25 (20%)	
**Attendance Frequency**	**Age Group**			
	**19–44**	**49–64**	**65 and Older**	***p*-Value**
2–3 times	21 (24%)	55 (27%)	60 (30%)	0.004
4–5 times	6 (7%)	21 (10%)	33 (16%)	
6–7 times	11 (13%)	15 (7%)	26 (13%)	
8–9 times	1 (1%)	7 (3%)	6 (3%)	
10–11 times	4 (5%)	12 (6%)	7 (3%)	
12+ times	9 (10%)	42 (21%)	34 (17%)	
**Attendance Frequency**	**Hispanic Origin**			
	**1–2 People**		**3 or More**	***p*-Value**
2–3 times	28 (31%)		108 (27%)	0.156
4–5 times	11 (12%)		49 (12%)	
6–7 times	6 (7%)		46 (12%)	
8–9 times	6 (7%)		8 (2%)	
10–11 times	3 (3%)		20 (5%)	
12+ times	12 (13%)		73 (18%)	
**Attendance Frequency**	**Food Security**			
	**High/Marginal**		**Low/Very Low**	***p*-Value**
2–3 times	64 (27%)		72 (29%)	0.911
4–5 times	32 (13%)		28 (11%)	
6–7 times	26 (11%)		26 (10%)	
8–9 times	8 (3%)		6 (2%)	
10–11 times	11 (5%)		12 (5%)	
12+ times	37 (16%)		48 (19%)	
**Attendance Frequency**	**SNAP/CalFresh/Food Stamps Participation**			
	**Yes**		**No**	***p*-Value**
2–3 times	30 (27%)		106 (28%)	0.565
4–5 times	10 (9%)		50 (13%)	
6–7 times	12 (115)		40 (11%)	
8–9 times	6 (5%)		8 (2%)	
10–11 times	5 (4%)		18 (5%)	
12+ times	20 (18%)		65 (17%)	
**Attendance Frequency**	**Number of Assistance Programs**			
	**None**	**1 Assistance Program**	**2 or More Assistance Programs**	***p*-Value**
2–3 times	37 (31%)	66 (27%)	33 (27%)	0.053
4–5 times	7 (6%)	39 (16%)	14 (11%)	
6–7 times	17 (14%)	20 (8%)	15 (12%)	
8–9 times	3 (3%)	3 (1%)	8 (6%)	
10–11 times	4 (3%)	14 (6%)	5 (4%)	
12+ times	18 (15%)	45 (18%)	22 (18%)	
**Attendance Frequency**	**Household Number**			
	**1 Household**		**2 or More Households**	***p*-Value**
2–3 times	79 (25%)		57 (32%)	0.624
4–5 times	36 (12%)		24 (13%)	
6–7 times	35 (11%)		17 (10%)	
8–9 times	9 (3%)		5 (3%)	
10–11 times	14 (5%)		9 (5%)	
12+ times	60 (19%)		25 (14%)	
**Attendance Frequency**	**House Size**			
	**1–2 People**		**3 or More**	***p*-Value**
2–3 times	33 (31%)		94 (27%)	0.710
4–5 times	12 (11%)		46 (13%)	
6–7 times	8 (7%)		40 (11%)	
8–9 times	2 (2%)		10 (3%)	
10–11 times	4 (4%)		18 (5%)	
12+ times	17 (16%)		64 (18%)	
**Attendance Frequency**	**Chronic Condition Count**			
	**None**	**1 Chronic** **Condition**	**2 or More** **Chronic Conditions**	***p*-Value**
2–3 times	22 (25%)	50 (31%)	53 (28%)	0.842
4–5 times	13 (15%)	20 (13%)	24 (13%)	
6–7 times	8 (9%)	15 (9%)	21 (11%)	
8–9 times	3 (3%)	5 (3%)	6 (3%)	
10–11 times	2 (2%)	5 (3%)	10 (5%)	
12+ times	12 (14%)	26 (16%)	35 (19%)	

### Appendix A.2

**Table A2:** Distribution of attendance frequency by produce consumption among returning users.

Attendance Frequency	Produce Consumed		
	All Produce Consumed	Less than All Produce Consumed	*p*-Value
2–3 times	76 (27%)	59 (37%)	0.001
4–5 times	40 (14%)	20 (12%)	
6–7 times	32 (11%)	20 (12%)	
8–9 times	9 (3%)	5 (3%)	
10–11 times	17 (6%)	5 (3%)	
12+ times	70 (25%)	15 (9%)	
